# Frequency-phase analysis of resting-state functional MRI

**DOI:** 10.1038/srep43743

**Published:** 2017-03-08

**Authors:** Gadi Goelman, Rotem Dan, Filip Růžička, Ondrej Bezdicek, Evžen Růžička, Jan Roth, Josef Vymazal, Robert Jech

**Affiliations:** 1 MRI Lab, The Human Biology Research Center, Department of Medical Biophysics, Hadassah Hebrew University Medical Center, Jerusalem, Israel; 2 Edmond and Lily Safra Center for Brain Sciences (ELSC), The Hebrew University of Jerusalem, Jerusalem, Israel; 3 Department of Neurology and Center of Clinical Neuroscience, First Faculty of Medicine and General University Hospital, Charles University in Prague, Prague, Czech Republic; 4 Department of Radiology, Na Homolce Hospital, Prague, Czech Republic

## Abstract

We describe an analysis method that characterizes the correlation between coupled time-series functions by their frequencies and phases. It provides a unified framework for simultaneous assessment of frequency and latency of a coupled time-series. The analysis is demonstrated on resting-state functional MRI data of 34 healthy subjects. Interactions between fMRI time-series are represented by cross-correlation (with time-lag) functions. A general linear model is used on the cross-correlation functions to obtain the frequencies and phase-differences of the original time-series. We define symmetric, antisymmetric and asymmetric cross-correlation functions that correspond respectively to in-phase, 90° out-of-phase and any phase difference between a pair of time-series, where the last two were never introduced before. Seed maps of the motor system were calculated to demonstrate the strength and capabilities of the analysis. Unique types of functional connections, their dominant frequencies and phase-differences have been identified. The relation between phase-differences and time-delays is shown. The phase-differences are speculated to inform transfer-time and/or to reflect a difference in the hemodynamic response between regions that are modulated by neurotransmitters concentration. The analysis can be used with any coupled functions in many disciplines including electrophysiology, EEG or MEG in neuroscience.

The last two decades have demonstrated that brain functionality and architecture can be better understood not only by identifying localized neural activity, but also, and perhaps primarily, by recognizing its connectivity. The distinction between functional segregation and integration and the use of network measures gave new means to understanding brain functionality. Within functional integration, two main classes of connectivity have emerged – functional (directed and undirected) and effective connectivity^1^. Functional connectivity refers to statistical dependencies amongst measured time-series, while effective connectivity rests on an explicit model of how those dependencies were caused (e.g., dynamic causal modelling^2^ and structural equation modelling^3^). In fMRI, coherent low frequency fluctuations of the blood-oxygenation-level dependent (BOLD) signal during a resting-state (rs-fMRI) were shown to contain functional neural network information^4^,^5^. This information was derived from the correlations between temporal fluctuations of BOLD signals in various brain regions in the absence of external stimuli^6^,^7^,^8^. Multiple resting-state networks were defined^6^,^9^,^10^ on the basis of such temporal correlations, and their reliability and robustness were shown at individual subjects and group levels^11^,^12^. These networks were shown to be in correlation with individual differences in behavioral performance^13^ and altered in neurological and psychiatric disorders^14^. Several computational models were proposed to link BOLD signal fluctuations to neuronal communication^15^,^16^,^17^.

Pearson’s correlation or independent component analyses (ICA) are the most commonly used methods to obtain the level of synchrony between time-series functions. However, these methods lack the ability to distinguish between different types of synchrony, such as those that are associated with different oscillation frequencies or those that are associated with a phase difference between the time-series. In here, we propose a new analysis method which enables to characterize functional connectivity by its frequencies and phases. The analysis is general and can be used in various data types and disciplines. In MRI, several approaches were introduced previously to obtain frequency-dependent functional connectivity. Those include coherences and partial coherences in frequency space^18^,^19^, undirected frequency dependent graphs^20^, spectral coherence matrix of pairwise interactions and cluster analysis^21^, mutual information in frequency space^22^,^23^ and recently nonlinear coherence between multiple time-series^24^. Other studies have included dynamic information by sliding-window analysis^25^,^26^,^27^, time-frequency analysis^28^, instantaneous phase synchronization^29^ or spontaneous coactivation patterns analysis^30^. Several studies have shown frequency-dependency of the BOLD signal and that this dependency is spatially dependent^23^,^31^. The observation that functional connectivity MRI is frequency dependent is supported by several findings. For example, in Parkinson’s disease patients the resting-state functional connectivity patterns of regions in the sensorimotor system were shown to differ between “OFF” and “ON” medication states^32^. Such differences are in line with known alterations of the frequencies in neuronal firing rates in those regions^33^. Furthermore, dependency of network topology on frequency^34^ was recently reported. The dependency of functional connectivity on phases (besides a phase of π) however, was not introduced before. In here, a method to observe functional connectivity with arbitrary phase-difference is introduced.

We present a unified framework for simultaneous assessment of frequency and latency of coupled time-series functions. We refer to latency as the phase difference between time-series functions and describe latency and functional connectivity by the same framework and as a function of frequency. Consequently, the latency is coupled in our analysis to the frequency and is represented by the phase-differences between a pair of time-series. The proposed analysis method transfers temporal 4D data (space and time) into a connectivity space in which each ‘functional connection’ (the relation between a pair of time-series) is represented by a cross-correlation with time-lag function. Using the general linear model, the weights of the time-series functions at specific frequencies and phase-differences are estimated. The strength of the proposed analysis is demonstrated on resting-state fMRI data of 34 healthy subjects. The main uniqueness of this method relative to other approaches considering frequency information in resting-state fMRI data^34^,^35^,^36^,^37^ is by providing means to characterize functional connections by their phase-differences. This allows identifying types of connections, anti-symmetric and asymmetric, that were not obtained by any other method. The paper focuses on the methodological description and not on neurobiological findings. We note however that the new functional connections contain important information, such as the functional connectivity of the cerebellum that has biological relevance.

## Mathematical description

The mathematical description below refers to resting-state functional connectivity MRI but can be modified to fit other types of coupled time-series functions in varies disciplines and particularly stimulus-driven fMRI, electrophysiology, Magnetoencephalography (MEG) or Electroencephalogram (EEG).

Assuming that the BOLD time-series signal can be approximated by a finite sum of weighted cosine and sine functions whose frequencies depend on the repetition time (TR) and the number of collected time points (N), it can be expressed as:


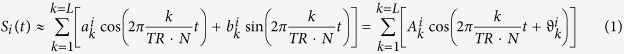


where *i* is a point in space (ROI or voxel), L is the number of functions used in the finite sum; 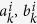 are the normalized weights of the cosine and sine functions respectively and L ≪ N due to the filter used in the preprocessing step. Equation 1 is simply the Fourier transform of the temporal signal where 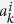 are the ‘real’ coefficients and 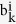 the ‘imaginary’ coefficients. The cross-correlation with time-lag function between two BOLD signals equals:


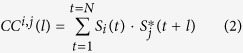


where *l* is the time-lag between the two BOLD signals; i,j are space indexes and ∗ denotes complex conjugate. Note that due to the normalization used in Equation 1, the Pearson’s correlation coefficient equals *CC*^*i, j*^(0). The frequency spectrum of Equation 2 equals:


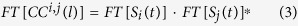


where FT denotes Fourier Transform. Combining Equations 1 and 3 provides an expression for the frequency spectrum of the cross-correlation function. Representing this function in the time domain (the cross-frequency terms are zero for orthogonal basis set) results with a new expression for the cross-correlation function:


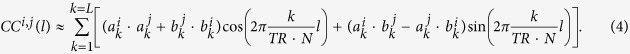


This expression implies that symmetric *CC*^*i, j*^(*l*) results from ‘in-phase’ weights, i.e., when both BOLD signals are either cosine or sine functions at the same frequency, antisymmetric *CC*^*i, j*^(*l*) results from ‘out-of-phase’ weights, i.e., when one BOLD signal is a cosine while the other is a sine function at the same frequency and asymmetric *CC*^*i, j*^(*l*) results from any other possible phase difference between the BOLD signals, i.e., when both symmetric and antisymmetric weights are significant. Note that the cross-wavelet transform^38^ and the wavelet transform coherence^39^ as well as our recent derivation^24^, give a similar expression to Equation 4 while using the wavelet space instead of Fourier space. Using Equation 4, the Pearson’s correlation coefficient is approximated by:


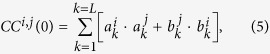


and therefore equals to the in-phase contributions of the cross-correlation function at time-lag zero.

To reduce the complexity of the analysis by assigning a small number of parameters (≪2 L), a general linear model (GLM) is applied to the *CC*^*i, j*^(*l*) functions (Equation 4). We chose frequencies for the GLM analysis which cover the entire frequency spectrum conventionally used for resting-state fMRI analysis (0.01–0.1 Hz). Discrete Fourier Transform theory indicates that the possible lowest frequency is 2*π*/(*N* · *TR*)=0.0104 Hz and the maximum number of terms needed to cover the frequency range is 8. We tested different numbers of basis set functions (from 4 to 8) and compared their agreement (goodness of the GLM fit) with Equation 4. A basis set of 8 functions: 4 cosines and 4 sines, was found to be appropriate. The following frequencies were selected: 0.02, 0.04, 0.06 and 0.08 Hz. Basis-set functions were multiplied by a window function. Three different window functions were tested and the Bartlett window was found to be best (see Supplementary Information Figure 1).

The GLM approach on the cross-correlation functions can therefore be written as:


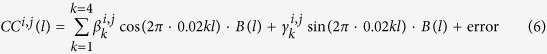


where *B(l*) is the Bartlett window function. Consequently, the new analysis termed hereinafter “Frequency-Phase Analysis” (FPA), results with eight scalars (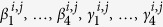) for each functional connection.

Equation 6 can be written to explicitly express the phase such that a functional connection with any possible phase can be obtained:


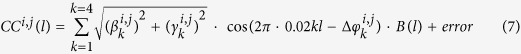


where


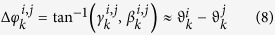


is the phase-difference between two BOLD signals, 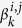 and 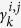 are given by Equation 6 and 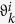 is given by Equation 1. Note that by using the GLM (Equation 6) to obtain functional connections with their phases, we avoid the need to apply statistics on complex numbers or directly on the phases which simplifies the analysis.

**Figure 1:**
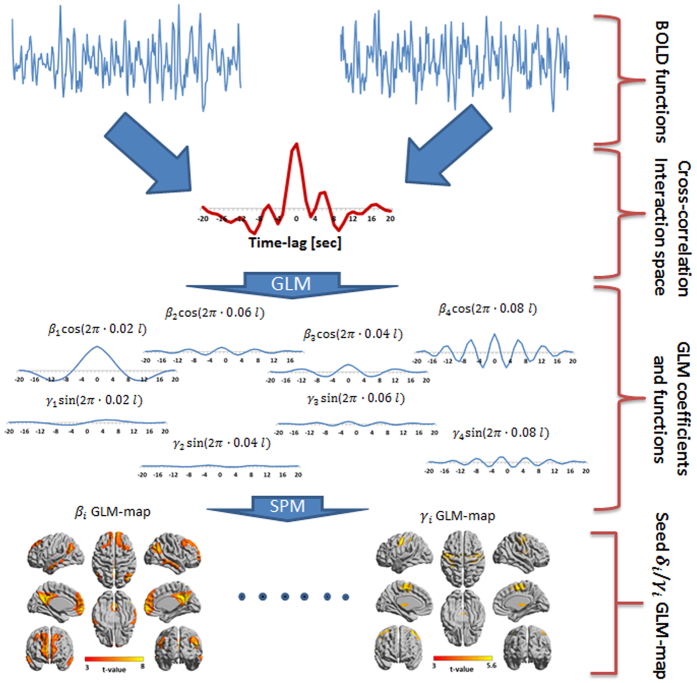
Flowchart diagram for the proposed analysis. Each pair of temporal time-series functions (e.g. BOLD signals) is cross-correlated to produce the cross-correlation function with time-lags. The cross-correlation functions define the “interaction space”. A general linear model (GLM) is then used with 8 basis set functions (4 cosine and 4 sine multiplied by the Bartlett window function) covering the entire frequency spectrum. The GLM results with 8 real values corresponding to the GLM weights. These values are used to construct seed GLM-SPMs for each weight. The example shown is for the interaction between the left precentral gyrus and the left putamen. For this example the strength of the interaction at frequency of 0.02 Hz is 0.08 with a phase of 8.4°; 0.02 for a frequency of 0.04 Hz with a phase of −9.2°; 0.017 for a frequency of 0.06 Hz with a phase of −31°; and 0.06 for a frequency of 0.08 Hz with a phase of −18°.

Figure 1 illustrates the proposed analysis method. For each pair of BOLD signals, a cross-correlation function is calculated (*CC*^*i, j*^(*l*)). This process transfers the 4D data (time and space) into “interaction space” that contains all pairwise cross-correlation functions. At the next stage, a GLM analysis is performed using a basis set of 4 cosine and 4 sine functions, each with a different frequency. The GLM analysis results with eight values that are the cosine and sine weights of the pairwise cross-correlation function. These values, for a pre-defined seed, are used to construct seed statistical parametric maps (SPMs) for each of the eight GLM weight (β/γ GLM-SPMs).

## Results

**Figure 2:**
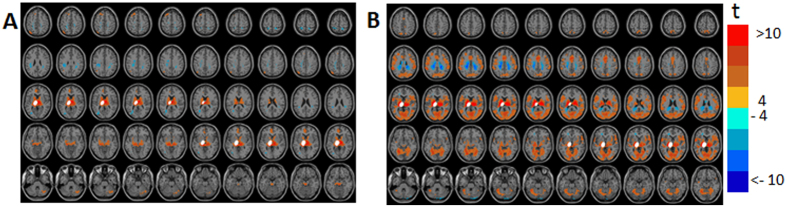
Seed-voxel statistical parametric maps (SPMs) of the left thalamus seed for two symmetric GLM-weights (GLM-SPMs). (**A**) β_1_ GLM-SPM (0.02 Hz). (**B**) β_4_ GLM-SPM (0.08 Hz). Voxels with significant GLM-weights (p < 0.01 Monte Carlo corrected for multiple comparisons) are shown in colors according to their t-value (the color bar is shown on the right). The thalamus seed is shown in white.

The *Frequency-Phase Analysis* (FPA) is applied here on resting-state fMRI data of 34 healthy subjects to demonstrate its ability to identify unique functional connections. Statistical parametric maps (SPMs) were calculated for each of the 8 GLM-weights. These maps, referred hereinafter as ‘GLM-SPMs’, allow to characterize the functional connectivity of seed regions according to their phases (β GLM-SPMs vs. γ GLM-SPMs) and frequencies (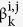 GLM-SPM vs. 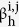 GLM-SPM). Figure 2 shows GLM-SPMs of the left thalamus for β_1_ and β_4_ GLM-weights (SPMs for all GLM-weights are shown in Supplementary Information Figure 2). In the figure, positive and negative t-values are indicated by red and blue colors, respectively, and the left thalamus seed is shown in white. The average (across all voxels and all subjects) F-value corresponding to the goodness of the GLM fit was 13.8 ± 0.006 (mean ± standard error), indicating high agreement between the GLM weights and cross-correlation functions in most of the voxels and demonstrating that 8 parameters are suitable to fit the cross-correlation functions. For the thalamus seed, almost no significant volumes were observed for the γ (antisymmetric) GLM-SPMs. GLM-SPMs of β_1–3_ (frequencies of 0.02, 0.04 and 0.06 Hz) were similar to each other but very different from β_4_ GLM-SPM (frequency of 0.08 Hz, Supplementary Information Figure 2). Note that in this figure (and other SPM figures below) negative GLM-weights are seen in the CSF, in white matter, around the ventricles, in large veins bordering CSF and in brain edges. These negative GLM-weights are thought to result from non-neuronal sources as was previously suggested^40^ and are ignored.

**Figure 3:**
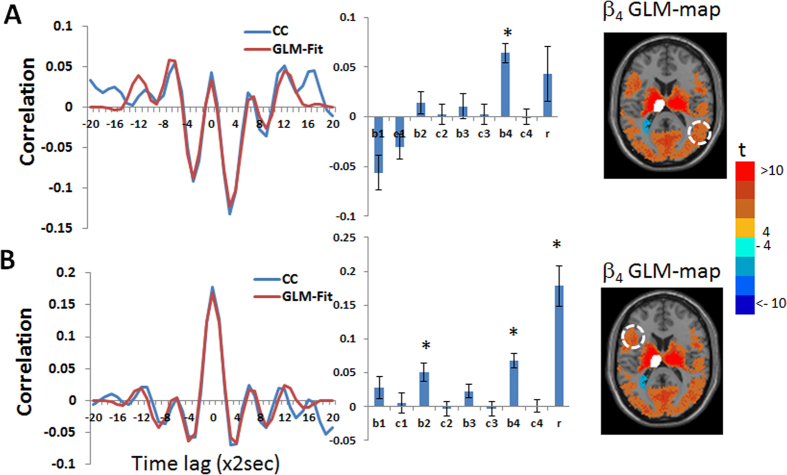
Group mean of cross-correlation functions, their GLM-fits and GLM-weights for the functional connectivity between the left thalamic seed and two cortical regions: occipital-temporal region (MNI = 42, −74, 16) and inferior frontal gyrus region (MNI = −50, 14, 16). The inserted images (from Fig. 2B) show β_4_ GLM-SPM of the left thalamus seed that contains the selected cortical regions with colors corresponding to t-values. The two cortical regions (temporal, frontal) are marked by white dashed lines on the image and the thalamus seed is shown in white. Betas 1-4 are denoted by b1-4, gammas 1-4 by c1-4, Pearson’s correlation coefficient by r and significant GLM-weights by*. Error bars correspond to the group standard errors. Group averaged cross-correlation (CC) functions are shown in blue and their GLM-fits (GLM-Fit) in red. (**A**) Cross-correlation between BOLD signals of the left thalamus and right temporal cortical region in which only *β*_4_ was found to be significant. (**B**) Cross-correlation between BOLD signals of the left thalamus seed and left frontal cortex region in which *β*_2_, *β*_4_ and Pearson’s correlation coefficient were found significant.

Figure 2 demonstrates the differences between β_1_ GLM-SPM and β_4_ GLM-SPM: while the functional connectivity of the left thalamus with voxels within the bilateral thalamus was manifested by all frequencies (i.e., significant for all betas), the left thalamus → occipital, cingulate, temporal and sensorimotor cortex functional connections were characterized only by the highest frequency (i.e., significant only for β_4_). To explore these differences, we calculated the functional connectivity (cross-correlation with time lag) between the left thalamus seed and two preselected regions. These regions were selected based on the GLM-SPMs according to their significant connectivity with the seed. Specifically, we calculated the following: (i) average cross-correlation functions, (ii) average GLM-weights (betas and gammas) and (iii) average Pearson’s correlation coefficients. All these were done across all voxels in the chosen regions, for each subject separately and presented as the group mean ± standard error (N = 34). The fitted GLM functions for the group mean cross-correlation functions (using Equation 6) are also shown. Figure 3 shows these calculations for the functional connectivity between the left thalamus and two regions in the occipital-temporal cortex and inferior frontal gyrus, indicated by white circles in the figure. Figure 3A shows the cross-correlation function between the left thalamus and a region in the occipital-temporal cortex (centered at MNI = 42, −74, 16). The cross-correlation function exhibits a significant symmetric high frequency component in addition to a tendency for significance of a symmetric and an antisymmetric low frequency components. Note, that in this case β_4_ is significant and β_1_ and γ_1_ show a tendency for significance while the Pearson’s correlation coefficient is not significant. Figure 3B shows the cross-correlation function between the left thalamus and a region in the inferior frontal gyrus (centered at MNI = −50, 14, 16). This functional connection was significant both by Pearson’s correlation coefficient and by the FPA. As seen, all γ (antisymmetric) GLM-weights are close to zero and Pearson’s correlation coefficient can be approximated by the sum of the β (symmetric) values, in line with Equation 5. Note the good agreement between the cross-correlation functions and their GLM-fits.

**Figure 4:**
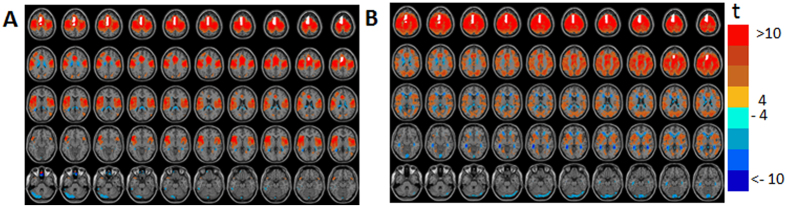
Seed-voxel statistical parametric maps (SPMs) of the left supplementary motor area (SMA) for two symmetric GLM-weights (GLM-SPMs). (**A**) β_1_ GLM-SPM (0.02 Hz). (**B**) β_4_ GLM-SPM (0.08 Hz). Voxels with significant GLM-weights (p < 0.01 Monte Carlo corrected for multiple comparisons) are shown in colors according to their t-value (color bar is shown on the right). The SMA seed is shown in white.

**Figure 5:**
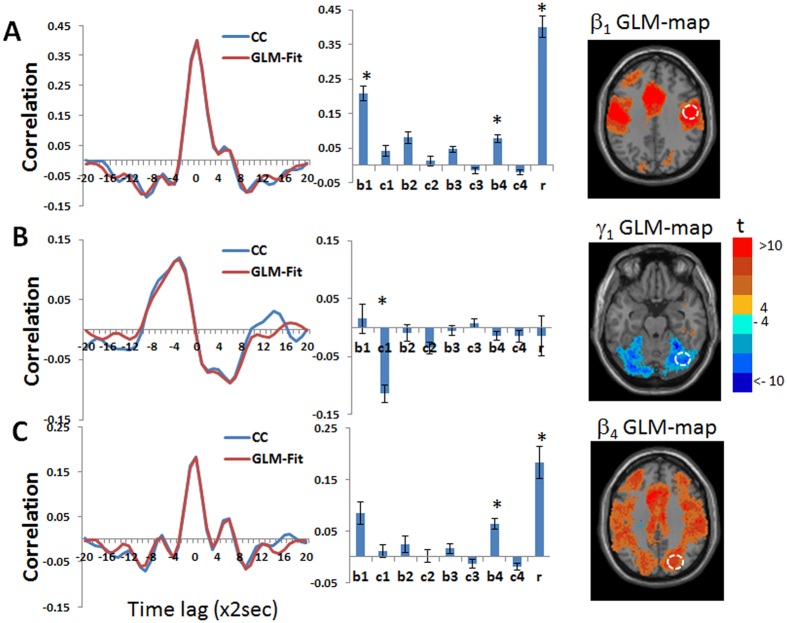
Group mean of cross-correlation functions (CC), their GLM-fits and GLM-weights for the functional connectivity between the left supplementary motor area (SMA) seed and three regions. Inserted images are from Fig. 4 and Supplementary Information Figure 3. Colors in the images correspond to t-values and the regions are marked by white dashed lines on the images. Group averaged cross-correlation (CC) functions are shown in blue and their GLM-fits (GLM-Fit) in red. Betas 1-4 are denoted by b1-4, gammas 1-4 by c1-4, Pearson’s correlation coefficient by r and significant GLM-weights by*. Error bars correspond to group standard errors. (**A**) Cross-correlation between the left SMA and a region in the right precentral cortex (MNI = 48, −16, 36). In the cross-correlation, *β*_2_, *β*_4_ and Pearson’s correlation coefficient were found significant. In the right is the β_1_ GLM-SPM containing the selected cortical region. (**B**) Cross-correlation between the left SMA and a region in the right posterior cerebellum (MNI = −32, −76, −24). In the cross-correlation, γ_1_ was found significant. In the right is the γ_1_ GLM-SPM containing the selected region. (**C**) Cross-correlation between the left SMA and a region in the right precuneus (MNI = 48, −16, 36). In the cross-correlation, *β*_4_ and Pearson’s correlation coefficient were found to be significant. In the right is the β_4_ GLM-SPM containing the selected region.

Figure 4 and Supplementary Information Figure 3 show GLM-SPMs of the left supplementary motor area (SMA) seed as an example for cortical GLM-SPMs. Average (across all voxels and all subjects) F-value was 16.01 ± 0.009 (mean ± SE). For this cortical seed, most of significant connections correspond to symmetric cross-correlation functions with positive weights. Antisymmetric connections are mainly with the cerebellum and at low frequencies. In the GLM-SPMs of the left SMA seed, SMA functional connectivity is mainly characterized by the lowest (β_1,_ Fig. 4A) and the highest (β_4_, Fig. 4B) frequencies which both show strong connectivity with the sensorimotor cortex. However, some differences are observed between β_1_ and β_4_ GLM-SPMs. For example, SMA-occipital and SMA-thalamic functional connectivity were significant only for the highest frequency (β_4_, Fig. 4B). Note that most of SMA-cerebellum connectivity is antisymmetric and at the lowest frequency (Supplementary Information Figure 3). Figure 5 shows the average (across voxels and subjects) of the GLM-weights, Pearson’s correlation coefficient, cross-correlation functions and GLM fits for the functional connectivity of the SMA with three different regions: a precentral region (centered at MNI = 48, −16, 36) (Fig. 5A), a cerebellar posterior region (centered at MNI = 32, −76, −24) (Fig. 5B) and a region in the precuneus (centered at MNI = 22, −74, 36) (Fig. 5C). These regions were selected from the GLM-SPMs based on their significant β_1_, γ_1_ and β_4_ weights respectively. For the SMA-precentral connectivity (Fig. 5A), β_1_, β_4_ and Pearson’s correlation coefficient values were significant. For the SMA-cerebellar connectivity (Fig. 5B), only the antisymmetric γ_1_ was significant. Note that the SMA-cerebellar connectivity corresponds to a time delay of ~12.5 sec (π/2 of a cycle with a frequency 0.02 Hz) in agreement with the maximum value of cross-correlation that is ~10 sec. For the SMA-precuneus connectivity (Fig. 5C), β_4_ and Pearson’s correlation coefficient values were significant. These three connectivity patterns represent the three different types of functional connections whose characterization is possible by the proposed analysis. Note again the good agreement between the cross-correlation functions and their GLM-fits, indicating the accuracy of the beta and gamma coefficients.

**Figure 6:**
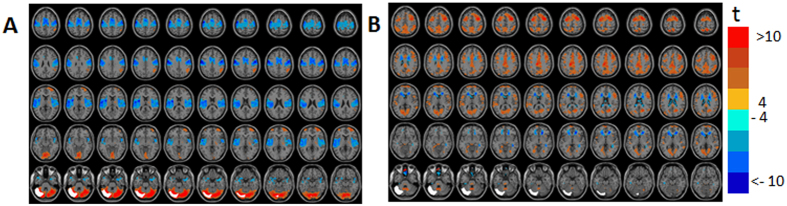
Seed-voxel statistical parametric maps (SPMs) of the left cerebellum crus 1 seed. (**A**) β_1_ GLM-SPM(0.02 Hz). (**B**) γ_1_ GLM-SPM (0.02 Hz). Voxels with significant GLM-weights (p < 0.01 Monte Carlo corrected for multiple comparisons) are shown in colors according to their t-value (color bar is shown on the right). The cerebellum crus 1 seed is shown in white.

**Figure 7:**
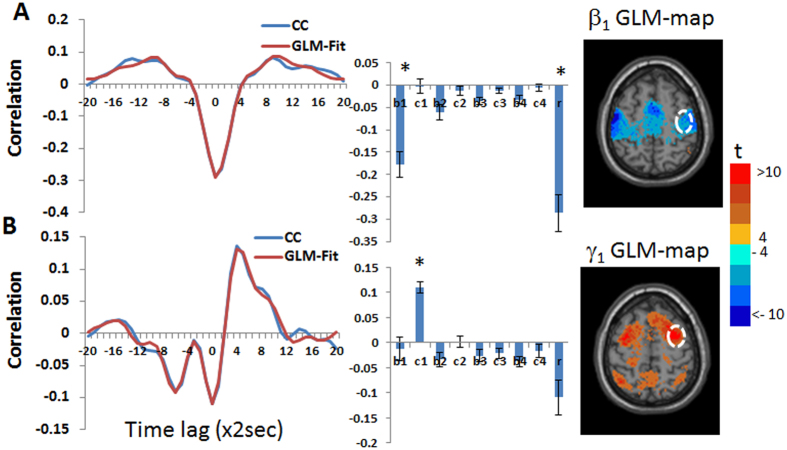
Group mean of cross-correlation functions, their GLM-fits and GLM-weights for the functional connectivity between the left cerebellum crus 1 seed and two cortical regions. Inserted images are from Fig. 6 with colors corresponding to t-values and the two cortical regions marked by white dashed lines. Group averaged cross-correlation (CC) functions are shown in blue and their GLM-fits (GLM-Fit) in red. Betas 1-4 are denoted by b1-4, gammas 1-4 by c1-4, Pearson’s correlation coefficient by r and significant GLM-weights by*. Error bars correspond to group standard errors. (**A**) Cross-correlation between the left cerebellum crus 1 and a region in the right precentral cortex (MNI = 38, −24, 56). In the cross-correlation, *β*_1_, *β*_4_ and Pearson’s correlation coefficient were found significant. In the right the β_1_ GLM-SPM containing the selected cortical region is shown. (**B**) Cross-correlation between the left cerebellum crus 1 and a region in the right frontal cortex (MNI = 24, −2, 56). In the cross-correlation, γ_1_ was found significant. In the right the γ_1_ GLM-SPM containing the selected region is shown.

Figure 6 and Supplementary Information Figure 4 show the GLM-SPMs of the left cerebellum crus 1 seed. The average (across all voxels and all subjects) F-value was 16.6 ± 0.008 (mean ± SE). For this seed, almost the entire functional connectivity was associated with the lowest frequency. In this low frequency range, both symmetric and antisymmetric GLM-SPMs had large, mostly not overlapping, connectivity volumes. The symmetric functional connectivity of the cerebellum crus 1 (β_1_ GLM-SPMs, Fig. 6A) was mainly with motor cortical areas (precentral gyrus) with negative weights, that corresponds to a time delay of ~25 sec since it is associated with a phase of π in a cycle of 0.02 Hz. The antisymmetric functional connectivity of the cerebellum crus 1 (γ_1_ GLM-maps, Fig. 6B) was with frontal, precentral, cingulate, SMA and occipital volumes with positive weights, that corresponds to a time delay of ~12.5 sec. Interestingly, almost all of the cerebellum crus 1 functional connectivity has a non-zero phase suggesting a significant time delay between cerebellar and cerebral activity. Figure 7 shows the average (across voxels and subjects) of GLM-weights, Pearson’s correlation coefficient, cross-correlation functions and their GLM-fits for the functional connectivity of the cerebellum crus 1 with two adjacent cortical regions: a precentral region (MNI = 38, −24, 56) and a frontal region (MNI = 24, −2, 56). For the cerebellar-precentral connectivity (Fig. 7A), β_1_ GLM-weight was significant and also the Pearson’s correlation coefficient, as expected since all antisymmetric weights were about zero. The cerebellar-frontal functional connection (Fig. 7B) demonstrates the unique type of antisymmetric connectivity with significant γ_1_ GLM-weight. Note that the sign of symmetric functional connections (such as SMA ↔ cerebellum) is independent of the order of calculation, while for antisymmetric functional connections the sign of the GLM-weights depends on the order of calculation. For example, connectivity of SMA → cerebellum (SMA is the seed) is negative while connectivity of cerebellum → SMA (cerebellum is the seed) is positive. This is due to the antisymmetric cross-correlation functions.

**Figure 8:**
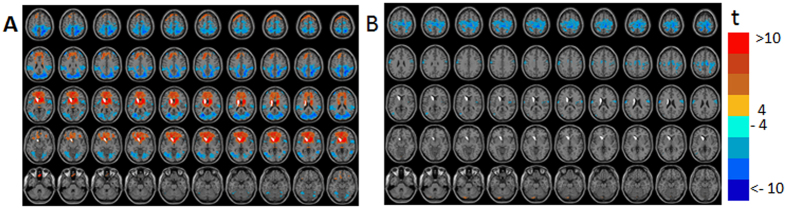
Seed-voxel statistical parametric maps (SPMs) of the left caudate seed. (**A**) GLM-SPM of β_1_ (0.02 Hz). (**B**) GLM-SPM of γ_1_ (0.02 Hz). Voxels with significant GLM-weights (p < 0.01 Monte Carlo corrected for multiple comparisons) are shown in colors according to their t-value (color bar is shown on the right). The caudate seed is shown in white.

**Figure 9:**
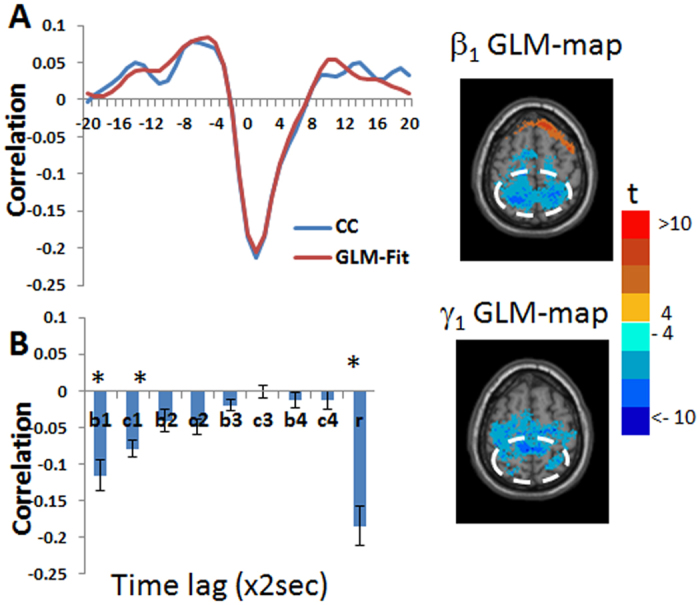
Group mean of cross-correlation functions, their GLM-fits and GLM-weights for the functional connectivity between the left caudate seed and voxels in the precuneus and paracentral lobule which show significance for both β_1_ and γ_1_ SPMs. Inserted images (from Fig. 8) show β_1_ and γ_1_ GLM-SPM of the same slice for the caudate seed that contains the selected voxels with colors corresponding to t-values. The white dashed lines present the area from which voxels were selected. (**A**) Group averaged cross-correlation (CC) function is shown in blue and its GLM-fit (GLM-Fit) in red. (**B**) GLM-weights. Betas 1-4 are denoted by b1-4, gammas 1-4 by c1-4, Pearson’s correlation coefficient by r and significant GLM-weights by*. Error bars correspond to group standard errors.

To demonstrate that the phase of a functional connection can be different from 0°, 90° or 180°, we chose post-hoc the caudate which demonstrates this type of functional connectivity, as a seed region. Figure 8 and Supplementary Information Figure 5 show the seed-SPMs of the left caudate. The average (across all voxels and all subjects) F-value was 10.03 ± 0.018 (mean ± SE). As shown, most of the caudate’s significant connections are at the lowest frequency with positive and negative symmetric GLM-weights in addition to many antisymmetric GLM-weights. Careful examination of Fig. 8 shows that at the lowest frequency certain voxels have both significant symmetric and antisymmetric GLM-weights. To test this further, we searched for voxels within the precuneus (MNI = 6, −54, 56) and paracentral lobule (MNI = 6, −40, 56) which were significant for both β_1_ and γ_1_ (symmetric and antisymmetric GLM-weights for the lowest frequency). Figure 9 shows the average ± SE (across voxels and subjects) of the GLM-weights, Pearson’s correlation coefficient, cross-correlation function and its GLM-fit for the connectivity of the caudate with these voxels. As seen, both β_1_ and γ_1_ are significant and the cross-correlation function shows a phase difference between the BOLD signals that according to Equation 8 equals to |34°| which corresponds to a time delay of 4.7 sec. This demonstrates that the proposed analysis can detect any possible phase-difference between a pair of BOLD functions.

## Discussion

In this paper we introduce a new resting-state functional connectivity analysis method which we name ‘Frequency-Phase Analysis’ (FPA). This analysis enables to characterize the functional connectivity between coupled time-series functions with respect to their frequencies and phases. It presents a simple unified framework for simultaneous assessment of the frequency and phase of functional connectivity under the assumption that the time-series can be expressed by Fourier series. Pairwise cross-correlation with time-lags of the time-series functions enables to infer coherent frequencies and phase-differences between the time-series. The mathematical framework allows to distinguish between symmetric, antisymmetric and asymmetric functional connectivity types. The general linear model (GLM) was used to obtain the weights of symmetric (cosine) and antisymmetric (sine) basis-set functions. These weights were used to calculate the phase-difference between time-series at specific frequencies (Equation 8). The frequency range and resolution of the cosine and sine basis-set functions was selected to cover the entire frequency spectrum and in accordance with the discrete Fourier transform theory. The excellent agreement between the GLM results and cross-correlation functions (the high F-values of the GLM-fits) suggest that the choice of the basis-set is adequate.

The Frequency-Phase Analysis is closely related to previous studies that use time-lags^41^,^42^,^43^,^44^,^45^. It was shown that the time-lag propagates in space within conventionally known resting-state networks, depends on neuronal state (e.g., eyes closed or open) and can be used to infer directionality of neural information flow^43^,^44^. On the other hand, clinical studies have identified vascular time-lags in patients suffering from hypoperfusion or ischemia^46^,^47^,^48^, suggesting a dependency of the time-lag on cerebrovascular perfusion. It therefore emerges that temporal delays can reveal time-lags of neural origin or reflect regional differences in vascular dynamics. Indeed, it was suggested that extracting neural propagating information from fMRI data is possible only when the variability of the hemodynamic response function is less than the time scale of information flow^49^. Efforts were made to characterize the differences between perfusion and neuronal contributions to the BOLD signal^45^ and to remove any confounds that can bias the time-lag analysis^50^,^51^. It is generally however accepted that for healthy subjects time-lags are related to neuronal transfer times with some neuro-vascular mapping, not entirely known. We claim that focusing on the time-lags could bias data interpretation and that instead the phases and phase-delays provide better measures of functional connectivity and information transfer. Since neuronal communication can be transmitted at certain frequencies and these neuronal frequencies are encoded in the fMRI signal^23^,^31^,^32^, times of information transfer do not necessarily coincide with the volume or efficiency of information transfer. Consequently, equal time-lags could correspond to different volumes of transferred information when they are transferred at different frequencies. For example, it was hypothesized that cortex-hippocampus functional connectivity during memory consolidation occurs at two different frequencies^44^. Consequently, a certain time-lag in this reciprocal process may correspond to different volumes of transferred information depending on the frequency in which it occurred. Thus, this suggests that phases or phase-differences provide better measures for information flow or for transfer efficiency.

Our analysis is also related to the cross-wavelet transform^38^, wavelet transform coherence^39^ and dynamic coherence analysis of Yaesoubi *et al*.^28^, with the first two using coherences module and the latter calculating phases as well. However, the aims and implications of Yaesoubi *et al*. and our proposed analysis are different. Yaesoubi *et al*. focuses on time-frequency analysis for dynamic evaluation of resting-state data, whereas here we focus on frequency-phase analysis to characterize functional connections by their phase differences. We also aim to introduce an analysis method that is similar to conventional correlation analyses. For this reason, the GLM approach was selected as it is a common functional MRI analysis approach. Besides its simplicity, the GLM approach used here reduces the number of parameters to 8 real numbers (the weights of the basis-set), which were shown to be sufficient to obtain good fits for the cross-correlation functions. In addition, since we aim to characterize functional connections by their type of phase differences (symmetric, antisymmetric and asymmetric) and not to define their phases, t-statistics can be used on each of the 8 GLM weights separately, with no need for complex-numbers statistics (e.g., circular Gaussian distributions or Gaussian hypergeometric distributions) which are more difficult and less familiar.

The ability of the FPA method to characterize functional connectivity by its phase-differences is demonstrated on resting-state fMRI data. We show that the FPA method identifies unique functional connections that were not defined before. Several examples are given for this attribute. Figure 3A shows functional connectivity of the left thalamus with a region in the occipital-temporal cortex, demonstrating an asymmetric type of functional connectivity that is not observed by the Pearson’s correlation analysis. This functional connection has a significant weight of a cosine function at 0.08 Hz along with close to significant weights of cosine and sine functions at 0.02 Hz. Their combined effect results with a phase-difference of ∼79° corresponding to a time delay of 10.9 sec. Since antisymmetric sine functions shift the cross-correlation function in time, the correlation value at time-lag zero (i.e., Pearson’s correlation) is reduced and insignificant. Figures 5B and 7B demonstrate examples in which Pearson’s correlation coefficients were not significant but the antisymmetric GLM-weights were significant. These connections were associated with cerebellar functional connectivity. An example for the ability of the FPA method to characterize connections by their dominant frequencies is given in Fig. 5A and C. These figures show functional connections which are significant by Pearson’s correlation analysis and by the FPA. However, the FPA method enables a more subtle characterization of these connections. Whereas the SMA-precentral functional connectivity (Fig. 5A) is mainly apparent in the lowest frequency, the SMA-occipital functional connectivity (Fig. 5C) is mainly apparent in the highest frequency. Figure 6 shows the GLM-SPMs of the left cerebellum crus 1 seed and demonstrates that antisymmetric functional connectivity can be associated with large gray matter volumes. Antisymmetric and symmetric functional connections in this figure correspond to anatomically different brain structures: the cerebellum crus 1 is functionally connected with the motor system by negative symmetric cross-correlation functions, and connected with frontal regions by antisymmetric cross-correlation functions.

Our interpretation of the biological meaning of fMRI time-series frequencies and phases is based on the assumption of a coupling between neuronal activity and BOLD signal and that the communication between different regions can be differently weighted by different frequencies^52^ and phases. Two principle mechanisms, applicable for healthy subjects, are presented below to explain the biological meaning of the measured phase differences in fMRI time-series. One is that phase-differences correspond to neuronal delays and reflect time of information transfer. This assumption was used by others to explain directionality in functional and effective connectivity^1^ and was used by us to show directionality of information flow and temporal organization using nonlinear coherence in wavelet space^24^. The second mechanism is related to the hemodynamic effect as we previously suggested^53^. We proposed that the hemodynamic responses of BOLD signals at different brain locations can be modulated by different weights. Specifically, if two remote regions with synchronous neuronal activity have different hemodynamic response functions, such that one is mainly affected by regional cerebral blood flow (rCBF) while the other is mainly affected by regional cerebral blood volume (rCBV), a time-delay between the two BOLD signals will be generated regardless of their neuronal coupling, since changes in rCBV are known to be delayed compared to changes in rCBF^54^,^55^,^56^. Consequently, one of the BOLD signals can, for example, be described by a cosine function with a zero phase, while the other by a cosine function at the same frequency with a non-zero phase. Such scenario may also occur when two regions are affected by different neurotransmitters or by different concentrations of neurotransmitters, since neurotransmitters are known to affect blood circuitry^53^.

### Conclusions

We introduce a new analysis method which characterizes functional connectivity between coupled time-series functions based on their frequencies and phase differences. We speculate that these phase differences in fMRI signals correspond to time of information transfer and/or to regional dependent hemodynamic effects with are modulated by neurotransmitters concentrations. Clearly, much more studies are needed to better understand the capabilities and physiological correlates of the new proposed analysis.

## Experimental Method

### Human Subjects and MRI acquisition

The study was approved by the Ethics Committee of the General University Hospital in Prague, Czech Republic. All subjects provided written informed consent for participation in the study and all methods were performed in accordance with the relevant guidelines and regulations. Magnetic resonance images were acquired with a 3T MR scanner (Magnetom Skyra, Siemens, Germany). Each participant underwent 10-minute resting-state functional MRI during which they were instructed to fixate on a visual crosshair, remain still and awake. Wakefulness was monitored during the whole scan using an MRI compatible camera. Functional images were acquired using T2*-weighted gradient EPI sequence with TR/TE 2 sec/30 ms, 300 repetitions and voxel size of 3 × 3 × 3 mm. 34 healthy subjects (age 64.9 ± 8.2, 18 men and 16 women) were included in the study. High resolution anatomical images were acquired using a sagittal T1-weighted magnetization-prepared rapid acquisition gradient echo (MP-RAGE) sequence for coregistration and normalization of the functional images to MNI space.

### Preprocessing of fMRI data and Pearson’s correlation analysis

Resting-state fMRI data was first preprocessed using Statistical Parametric Mapping (SPM8, Welcome Trust Centre for Neuroimaging, London, United Kingdom, http://www.fil.ion.ucl.ac.uk/spm/software/spm8). Standard preprocessing steps included: realignment, coregistration, normalization to MNI-space and spatial smoothing with an 8 mm Gaussian kernel. Voxels of 2 × 2 × 2 mm^3^ were obtained after these steps. An a-priori standard inclusion criterion of maximal head motion <3 mm or 3° rotations was chosen although the average displacement for subjects was <1.5 mm and <1.5° rotation. Functional connectivity analysis of resting-state data was done using CONN toolbox^57^. Further preprocessing steps included: removal of confounds by regression (motion parameters, first principal components of CSF and white matter signals), linear detrending and band-pass filtering (0.01–0.1 Hz). Removal of the first principle components of the CSF and white matter signals by regression was used rather than removal of global signal in order to minimize biases that might be introduced by global signal regression^40^,^58^,^59^,^60^,^61^. Scrubbing was further done using DPARSF toolbox^62^. Regions of interest (ROIs) for seed analysis were selected using the Automated Anatomical Labeling (AAL)^63^. Although some AAL regions can be further sub-divided to more specific functional regions (e.g. the thalamus), standard AAL regions were chosen to demonstrate different functional connectivity between cortical, subcortical and cerebellar regions.

### GLM of cross-correlation functions

All further calculations were performed with IDL version 6.1 using custom-developed software. Cross-correlation functions were generated using 41 time-lag points which correspond to a range of −40 to 40 seconds. This time range was chosen to include all relevant basis-set frequencies (0.02–0.08 Hz) and to cover the relevant length of the post-stimulus response expected to modulate the hemodynamic response function of the BOLD signal by regional cerebral blood volume (rCBV)^64^. To study possible effects of lower frequencies (<0.02 Hz), a larger range of time-lag points should be selected. Beta and gamma values, i.e., the coefficients of the cosine and sine basis-set functions of Equation 6, were calculated using standard IDL routine for linear regression. Consequently, the FPA method generated 8 × N scalars for each seed: 4 beta and 4 gamma values for each of the N functional connections.

### Statistics

The level of significance in the FPA GLM-SPM calculations was defined by one sample t-test on each GLM weight separately. Significance was defined by a combination of a voxel-level p-value of p < 0.0001 and a cluster-level threshold of 100 voxels. The cluster size was chosen according to Monte-Carlo simulation to set the threshold at p < 0.01 corrected for multiple comparisons^65^. For the ROI statistics (Figures 3, 5, 7 and 9), one sample t-test on each GLM weight was done separately using a threshold of p < 0.0001.

### Choice of seed regions

Seed-voxel statistical-parametric-maps (SPM) were obtained from 3 predefined ROIs that represent different types of brain regions (cortex, subcortex and cerebellum) and are all part of the motor system. These were: the left supplementary motor area (SMA), left thalamus and left cerebellum crus 1. The motor system was chosen arbitrarily as an example to demonstrate the capabilities of the proposed analysis. At a later stage, we searched for clusters in the GLM-SPM that showed asymmetric functional connectivity. The left caudate was identified as showing asymmetric functional connections and therefore chosen as a 4^th^ seed region. Note that these seeds were not chosen by their goodness of fit to Equation 6.

## Additional Information

**How to cite this article:** Goelman, G. *et al*. Frequency-phase analysis of resting-state functional MRI. *Sci. Rep.* **7**, 43743; doi: 10.1038/srep43743 (2017).

**Publisher's note:** Springer Nature remains neutral with regard to jurisdictional claims in published maps and institutional affiliations.

## Supplementary Material

Supplementary Information
