# Corrigendum: Frequency-phase analysis of resting-state functional MRI

**DOI:** 10.1038/srep45803

**Published:** 2017-04-10

**Authors:** Gadi Goelman, Rotem Dan, Filip Růžička, Ondrej Bezdicek, Evžen Růžička, Jan Roth, Josef Vymazal, Robert Jech

Scientific Reports 7: Article number: 4374310.1038/srep43743; published online: 03 08 2017; updated: 04 10 2017

This Article contains errors in the Acknowledgements section.

“This work was supported by a Czech-Israel cooperative research grant issued by the Israel Ministry of science (grant number 3–9814) and the Czech Ministry of education (Grant LH13256 VES13-KontaktII). Additional support was given by the Czech Ministry of Health (Grant IGA MZ CR NT12282-5/2011) and the Charles University in Prague (Research Project PRVOUK-P26/LF1/4).”

Should read:

“This work was supported by the Czech Science Foundation (grant GACR 16-13323S) and by the Czech-Israel cooperative research grant (Israel Ministry of science: grant 3–9814, and Czech Ministry of education: grant LH13256 VES13-KontaktII)”.

