# Supplementary material for: Frequency-phase analysis of resting-state functional MRI
Source: Sci Rep. 2017 Mar 8;7:43743. doi: 10.1038/srep43743 (PMC5341062; doi:10.1038/srep43743)
Supplement: Supplementary Information [file srep43743-s1.doc]

**Supplementary Information for:**

**Title: Frequency-phase analysis of resting-state functional MRI**

Authors:

Gadi Goelman ^1*^

Rotem Dan^1,2^

Filip Růžička^3^

Ondřej Bezdíček^3^

Evžen Růžička^3^

Jan Roth^3^

Josef Vymazal^4^

Robert Jech^3^

^1^MRI Lab, the human Biology Research Center, Department of Medical Biophysics

Hadassah Hebrew University Medical Center, Jerusalem, Israel

^2^ Edmond and Lily Safra Center for Brain Sciences (ELSC), The Hebrew University of Jerusalem, Jerusalem, Israel

^3^Department of Neurology and Center of Clinical Neuroscience, First Faculty of Medicine and General University Hospital, Charles University in Prague, Prague, Czech Republic

^4^Department of Radiology, Na Homolce Hospital, Prague, Czech Republic

**
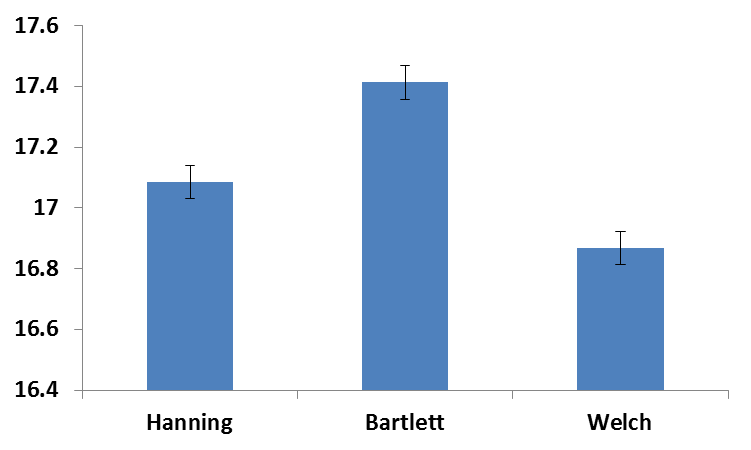
**

**F-value**

**SI Figure 1: The effect of window function in the regression process of Equation 6**

The goodness of GLM fit of equation 6 was tested for all cross correlation functions using three different window functions: the Hanning, Bartlett and the Welch. Cross correlation with time lag was calculated for BOLD signals of the 116 AAL ROIs for all subjects (116*115/2*34 calculations). For each regression and each window selection, the F-value was obtained and their mean ± standard errors are presented below. The Bartlett window function was found best and was use in the entire analysis.

**SI Figure 2: GLM SPMs of the left Thalamus seed**

Seed-voxel statistical parametric maps (SPMs) of the left thalamus seed (GLM-SPMs). (A) β_1_ GLM-SPM (0.02 Hz). (B) γ_1_ GLM-SPM (0.02 Hz). (C) β_2_ GLM-SPM (0.04 Hz). (D) γ_2_ GLM-SPM (0.04 Hz). (E) β_3_ GLM-SPM (0.06 Hz). (F) γ_3_ GLM-SPM (0.06 Hz). (G) β_4_ GLM-SPM (0.08 Hz). (H) γ_4_ GLM-SPM (0.08 Hz). Voxels with significant GLM-weights (p<0.01 Monte Carlo corrected for multiple comparisons) are shown in colors according to their t-value (color bar is shown on the right). The thalamus seed is shown in white.


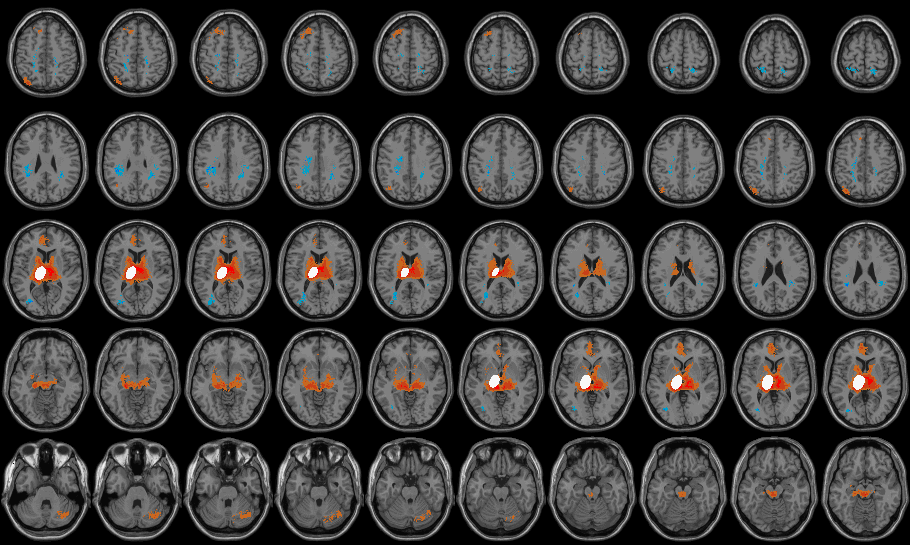

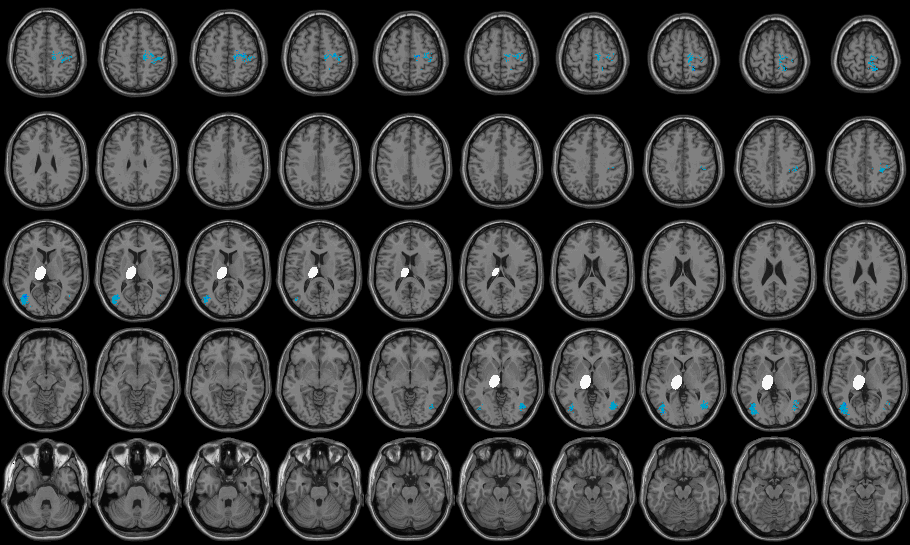

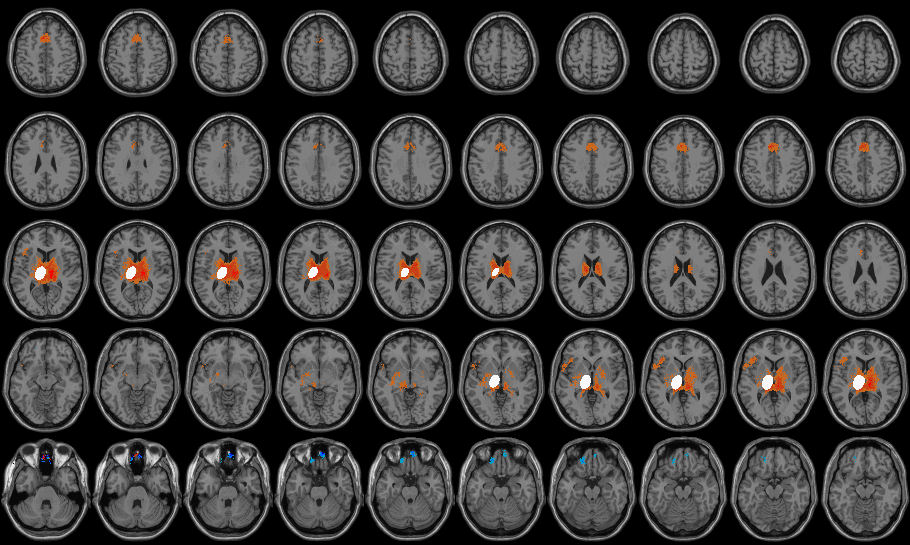

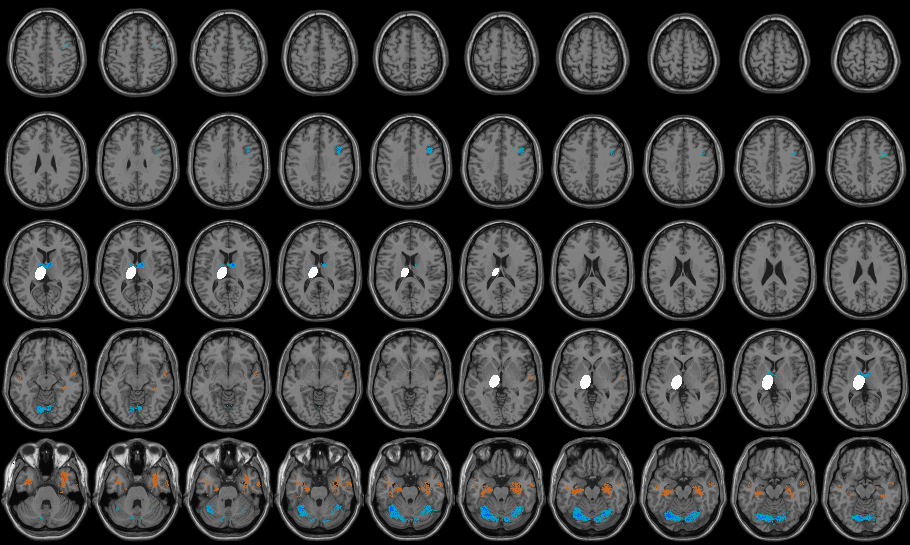

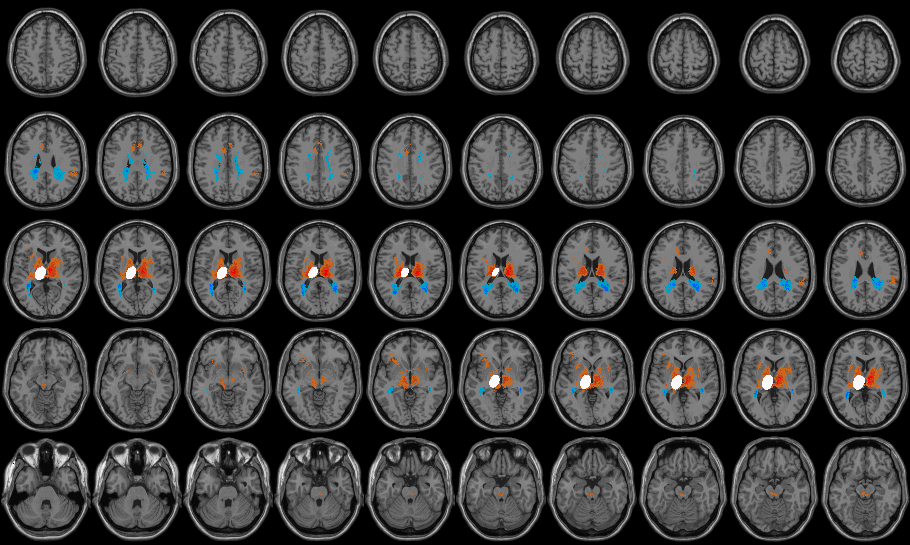

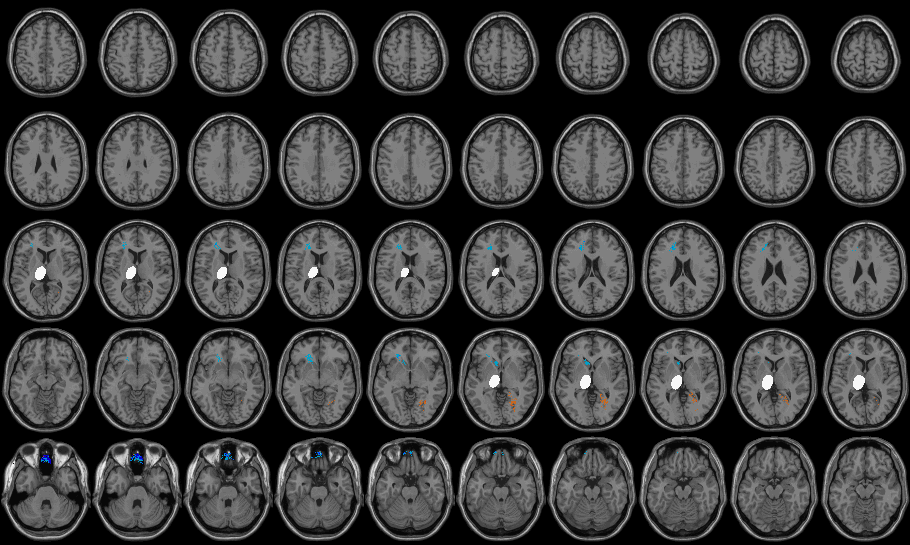

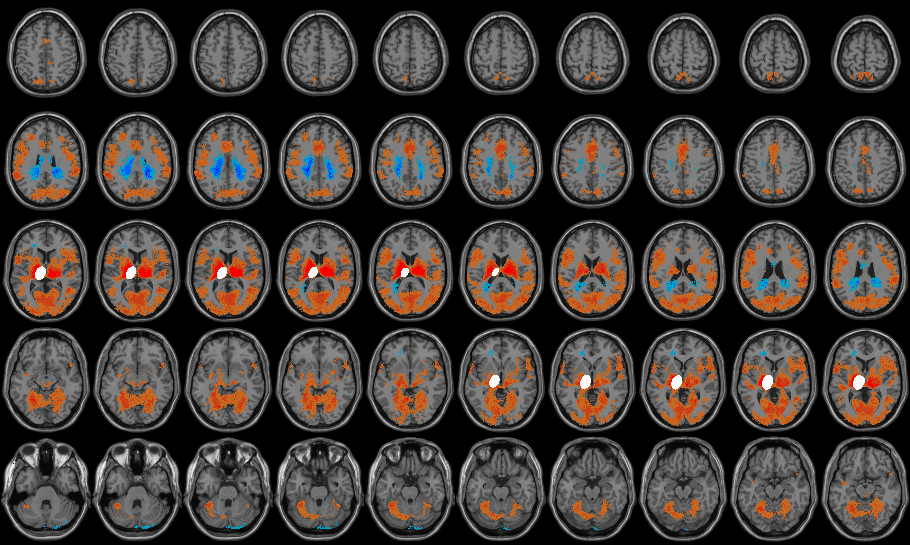

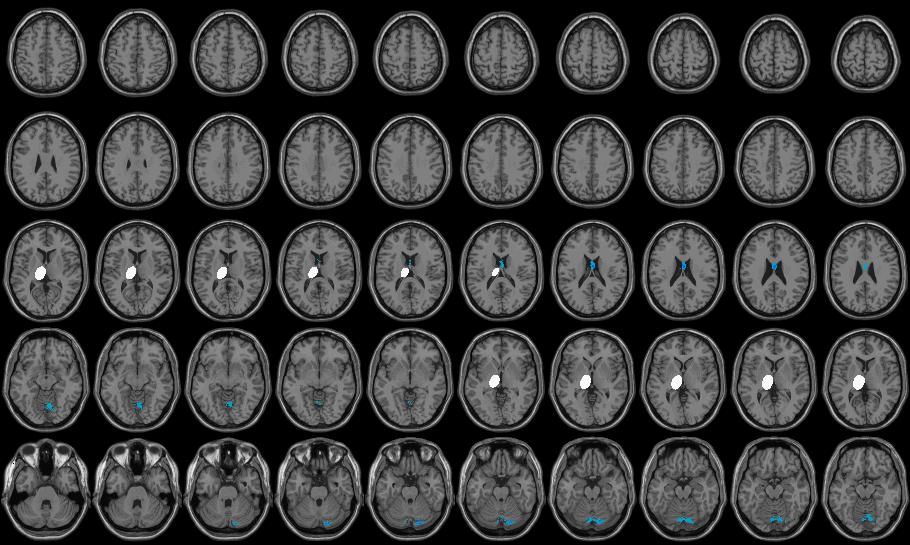

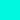

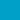

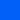

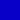

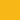

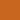

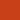

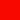


<- 10

- 4

4

>10

t

**A**

**C**

**E**

**G**

**B**

**D**

**F**

**H**

**SI Figure 3: GLM SPMs of the left Supplementary Motor Area (SMA) seed**

Seed-voxel statistical parametric maps (SPMs) of the left SMA seed (GLM-SPMs). (A) β_1_ GLM-SPM (0.02 Hz). (B) γ_1_ GLM-SPM (0.02 Hz). (C) β_2_ GLM-SPM (0.04 Hz). (D) γ_2_ GLM-SPM (0.04 Hz). (E) β_3_ GLM-SPM (0.06 Hz). (F) γ_3_ GLM-SPM (0.06 Hz). (G) β_4_ GLM-SPM (0.08 Hz). (H) γ_4_ GLM-SPM (0.08 Hz). Voxels with significant GLM-weights (p<0.01 Monte Carlo corrected for multiple comparisons) are shown in colors according to their t-value (color bar is shown on the right). The SMA seed is shown in white.


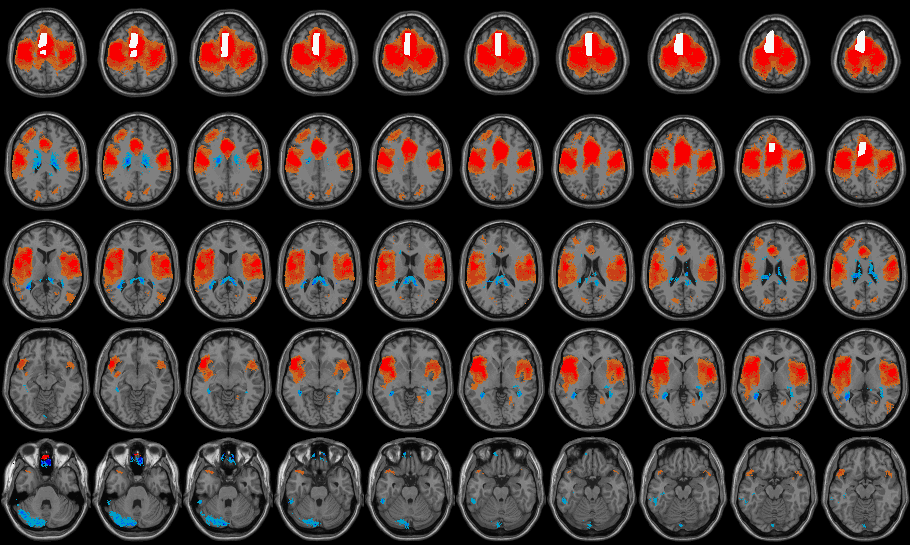

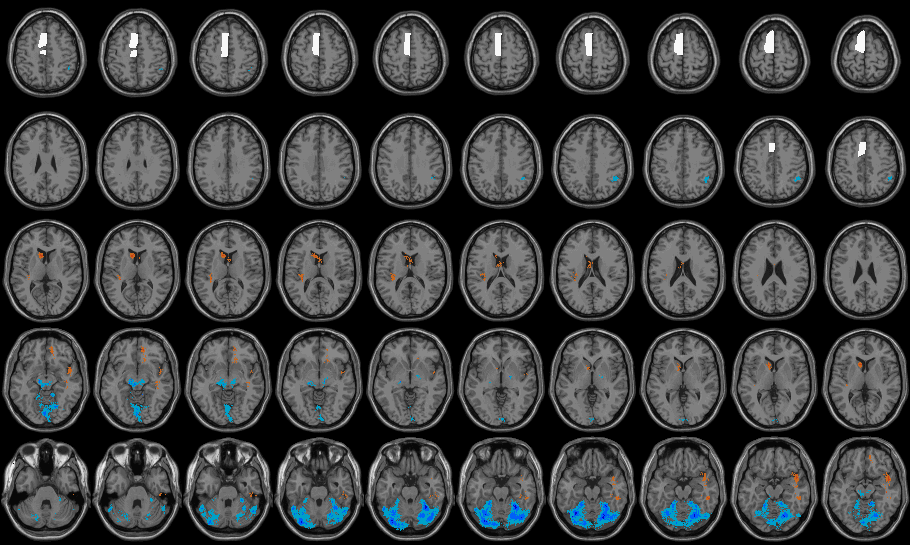

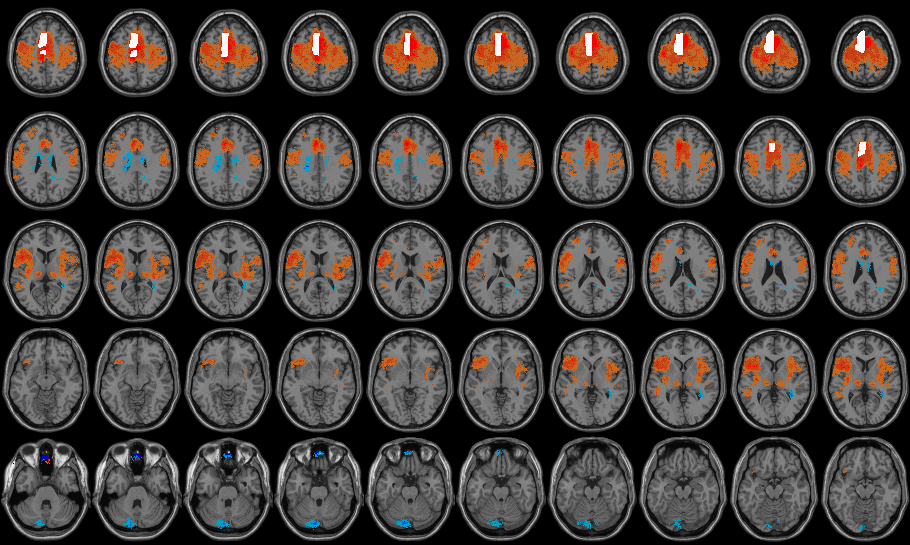

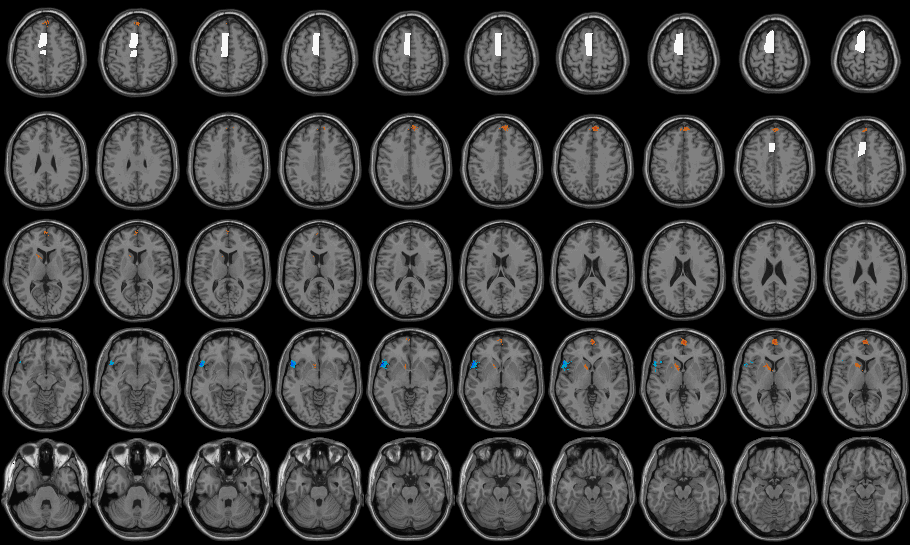

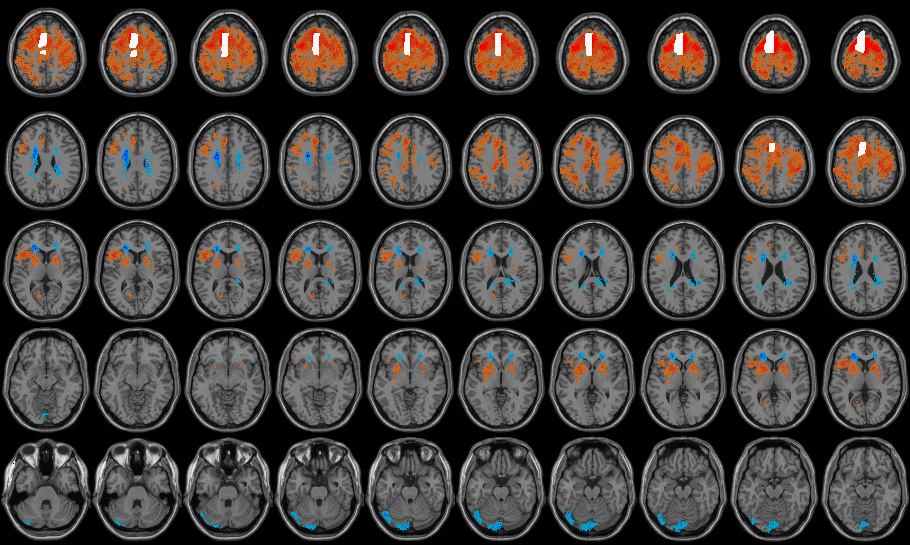

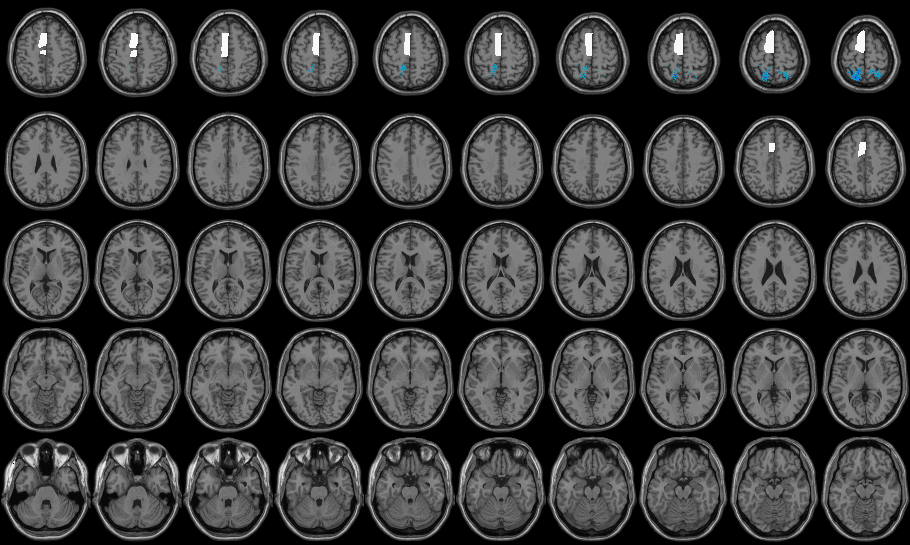

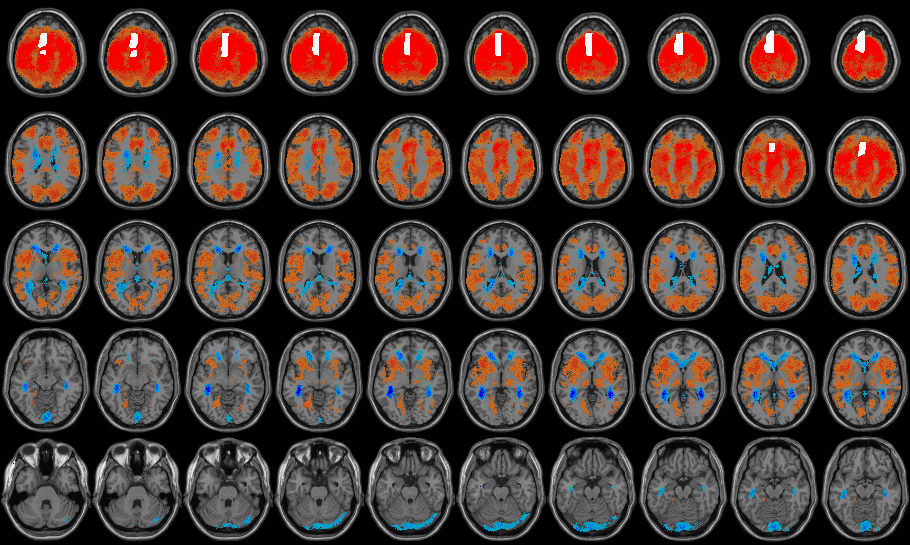

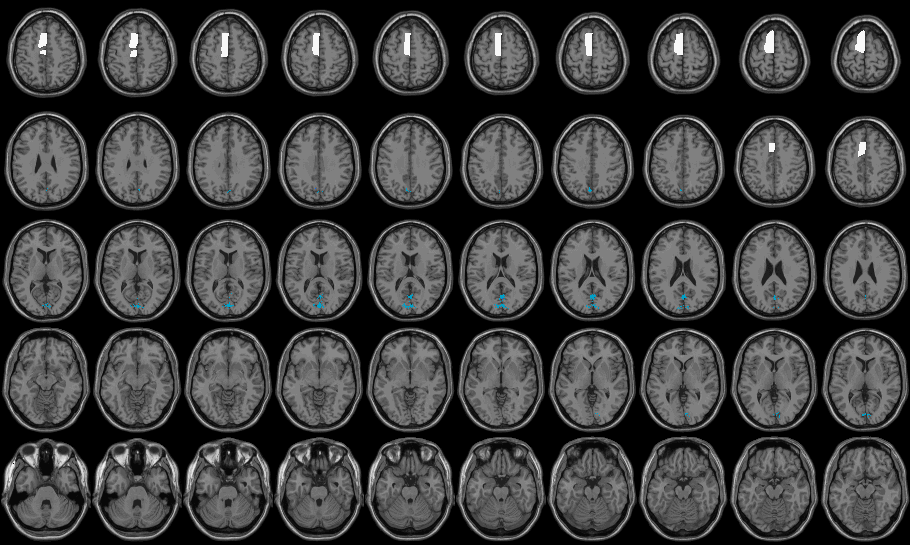

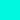

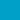

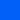

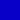

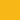

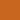

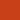

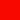


<- 10

- 4

4

>10

t

**A**

**C**

**E**

**G**

**B**

**D**

**F**

**H**

**SI Figure 4: GLM SPMs of the left Cerebellum Crus 1 seed**

Seed-voxel statistical parametric maps (SPMs) of the left Cerebellum Crus 1 seed (GLM-SPMs). (A) β_1_ GLM-SPM (0.02 Hz). (B) γ_1_ GLM-SPM (0.02 Hz). (C) β_2_ GLM-SPM (0.04 Hz). (D) γ_2_ GLM-SPM (0.04 Hz). (E) β_3_ GLM-SPM (0.06 Hz). (F) γ_3_ GLM-SPM (0.06 Hz). (G) β_4_ GLM-SPM (0.08 Hz). (H) γ_4_ GLM-SPM (0.08 Hz). Voxels with significant GLM-weights (p<0.01 Monte Carlo corrected for multiple comparisons) are shown in colors according to their t-value (color bar is shown on the right). The Cerebellum Crus 1 seed is shown in white.


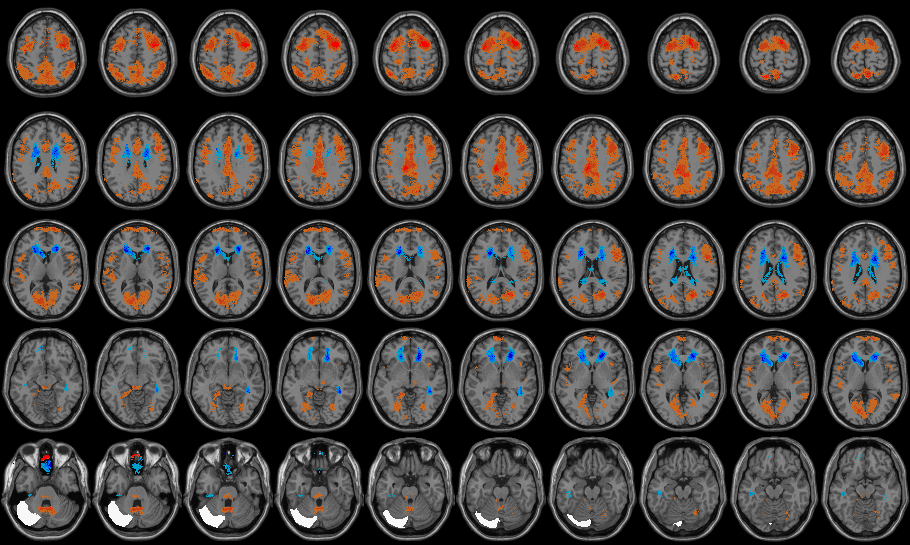

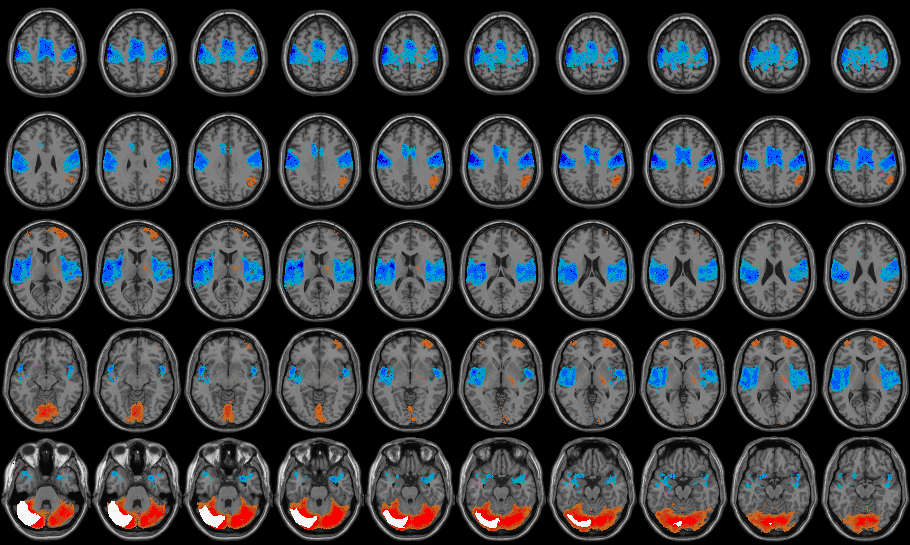

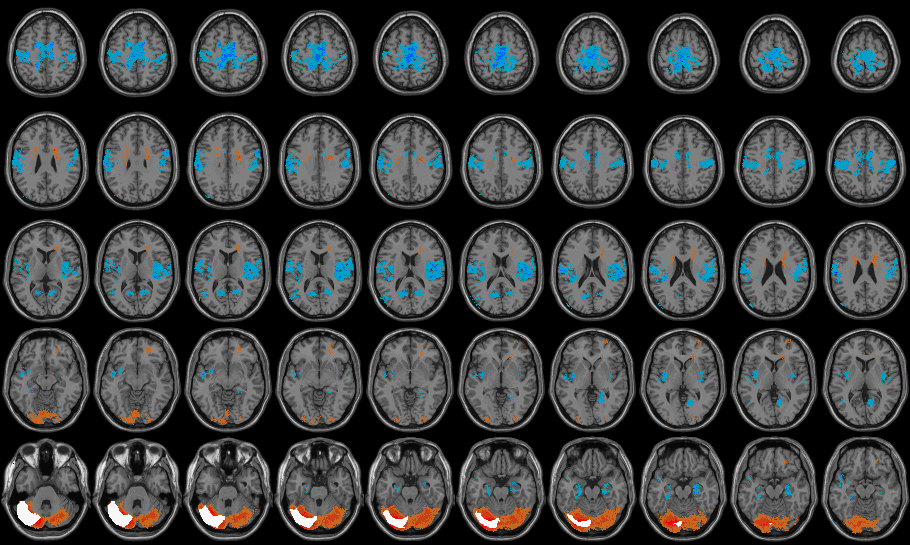

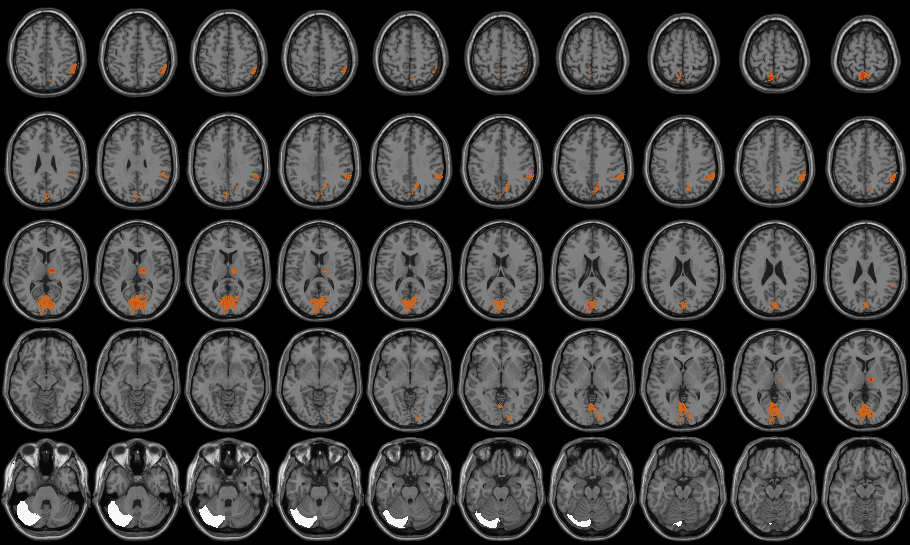

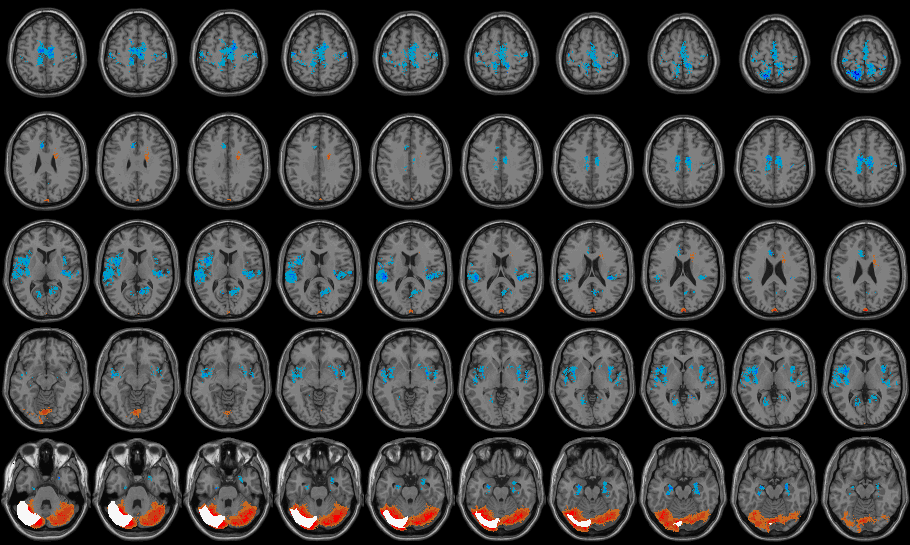

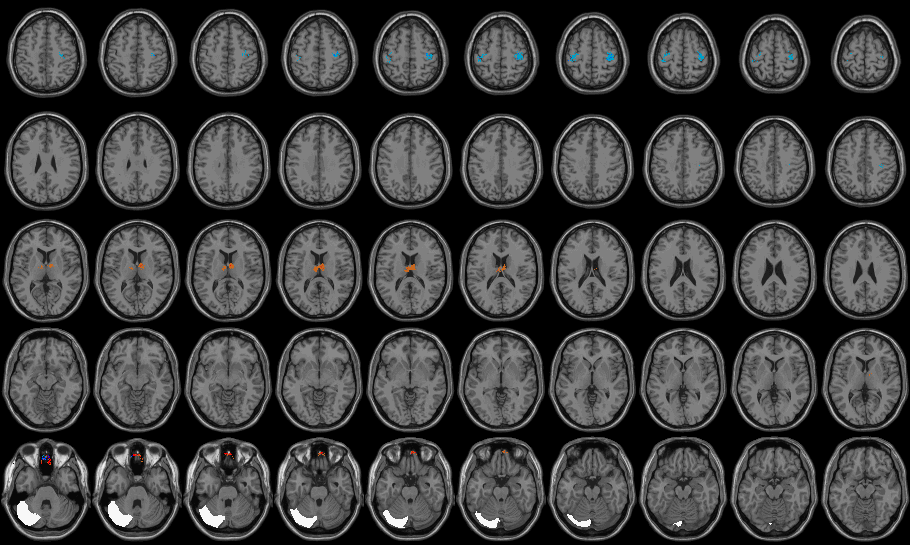

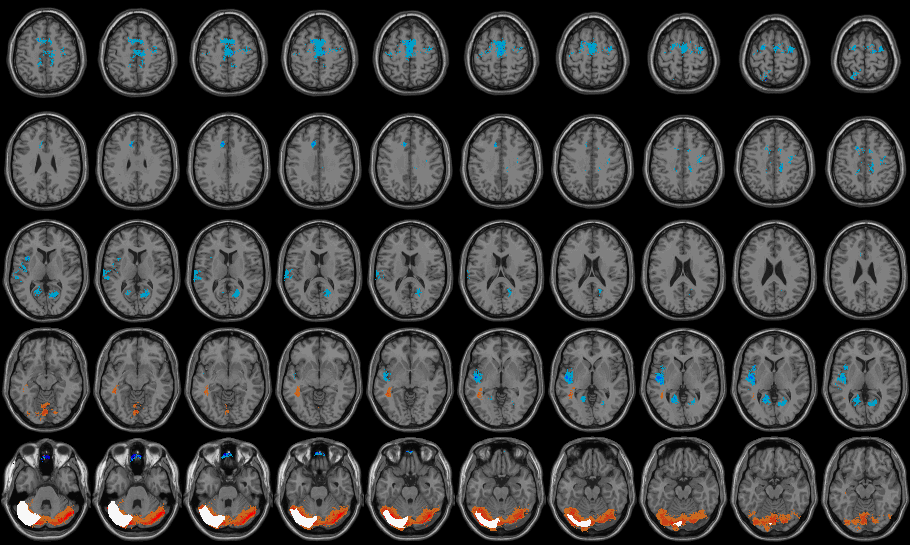

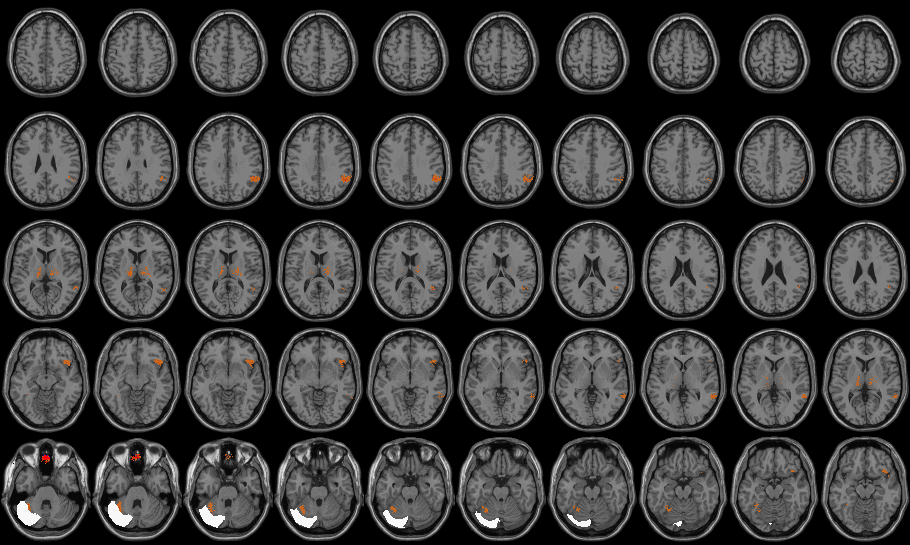

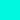

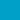

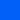

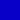

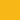

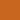

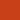

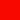


<- 10

- 4

4

>10

t

**A**

**C**

**E**

**G**

**B**

**D**

**F**

**H**

**SI Figure 5: GLM SPMs of the left caudate seed**

Seed-voxel statistical parametric maps (SPMs) of the left caudate seed (GLM-SPMs). (A) β_1_ GLM-SPM (0.02 Hz). (B) γ_1_ GLM-SPM (0.02 Hz). (C) β_2_ GLM-SPM (0.04 Hz). (D) γ_2_ GLM-SPM (0.04 Hz). (E) β_3_ GLM-SPM (0.06 Hz). (F) γ_3_ GLM-SPM (0.06 Hz). (G) β_4_ GLM-SPM (0.08 Hz). (H) γ_4_ GLM-SPM (0.08 Hz). Voxels with significant GLM-weights (p<0.01 Monte Carlo corrected for multiple comparisons) are shown in colors according to their t-value (color bar is shown on the right). The caudate seed is shown in white.


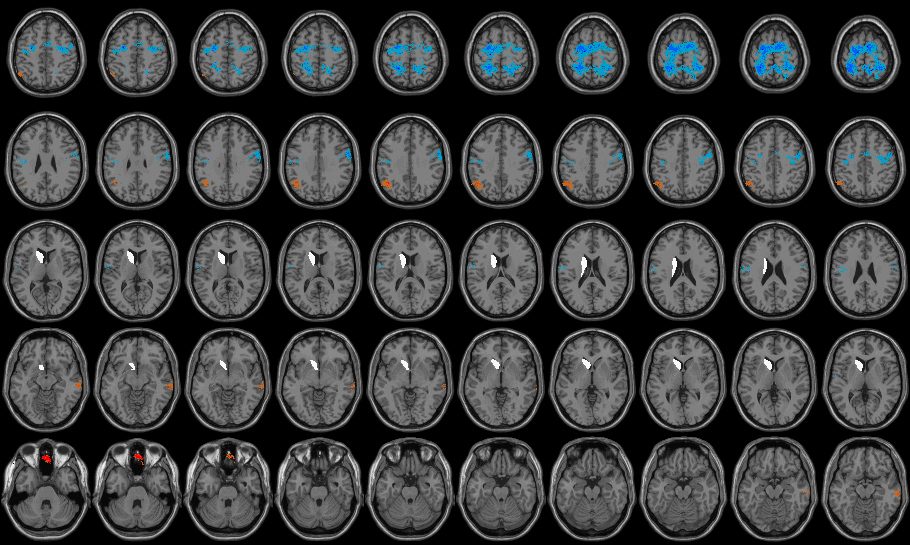

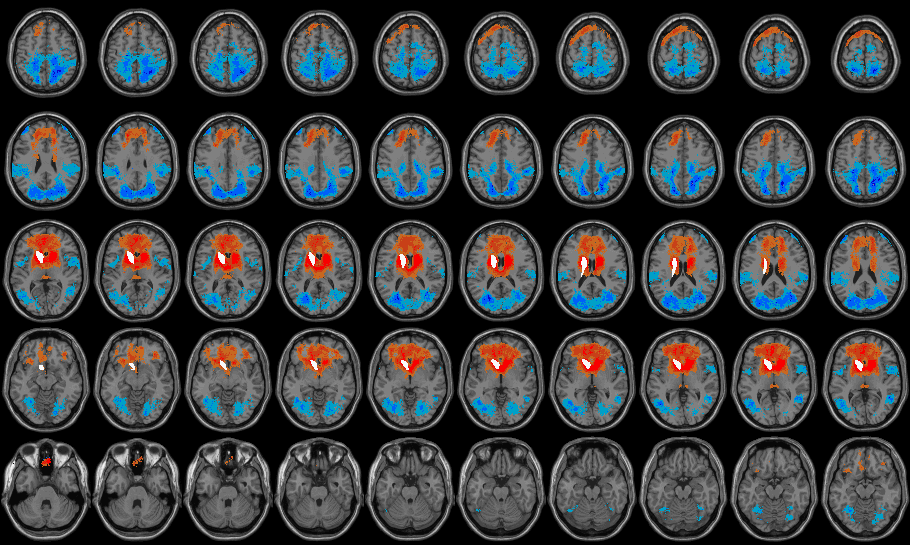

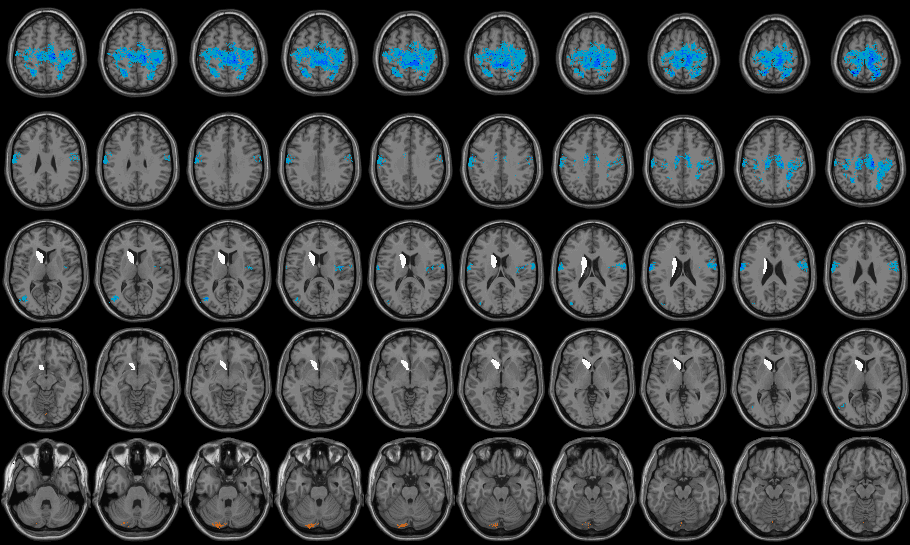

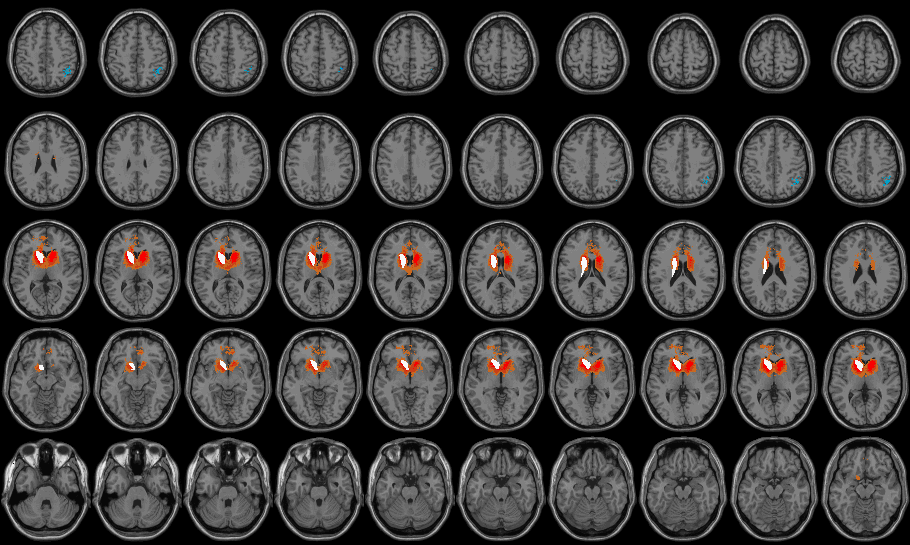

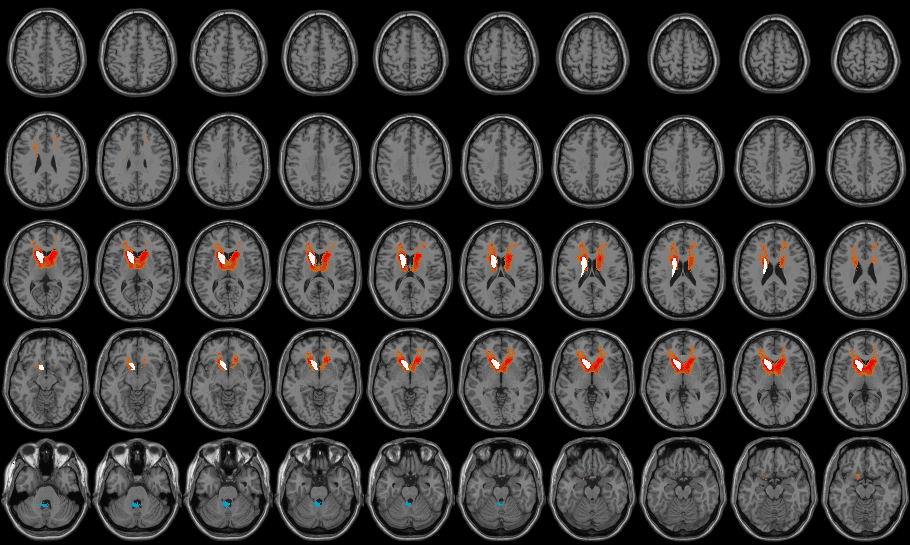

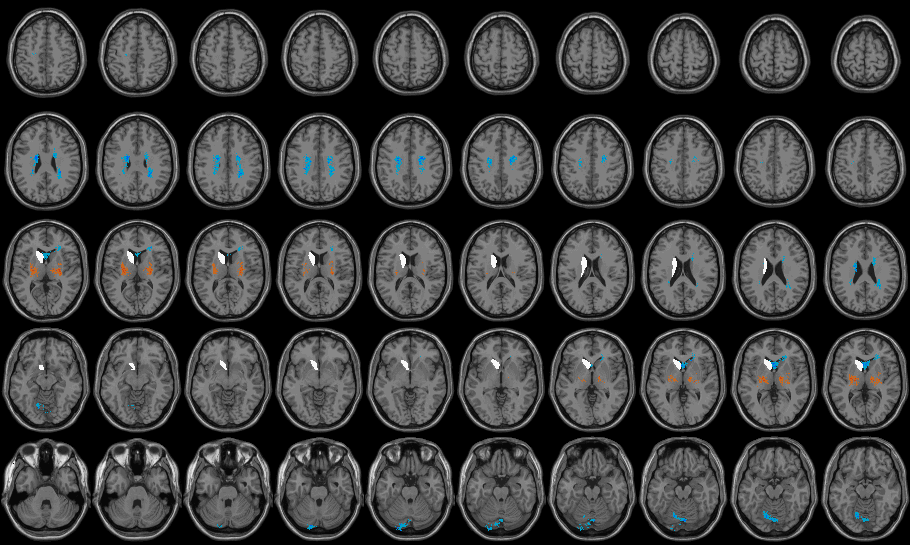

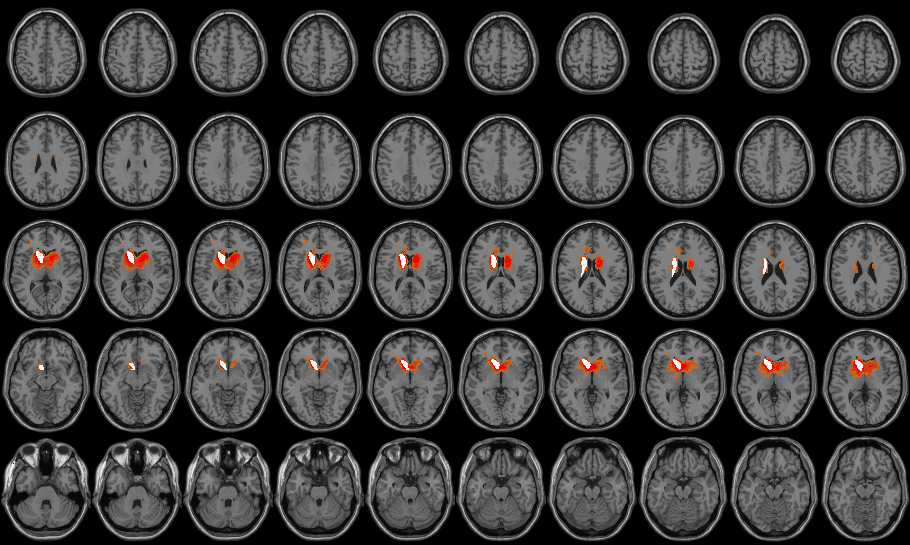

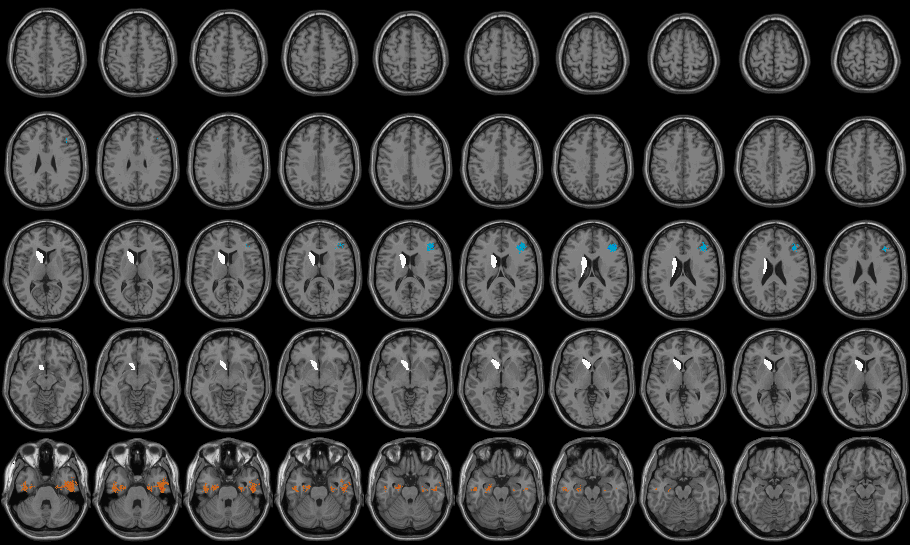


**A**

**C**

**E**

**G**

**B**

**D**

**F**

**H**


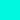

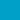

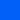

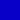

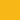

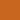

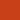

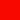


<- 10

- 4

4

>10

t
